# Artificial-Intelligence-Aided Radiographic Diagnostic of Knee Osteoarthritis Leads to a Higher Association of Clinical Findings with Diagnostic Ratings

**DOI:** 10.3390/jcm12030744

**Published:** 2023-01-17

**Authors:** Markus Neubauer, Lukas Moser, Johannes Neugebauer, Marcus Raudner, Barbara Wondrasch, Magdalena Führer, Robert Emprechtinger, Dietmar Dammerer, Richard Ljuhar, Christoph Salzlechner, Stefan Nehrer

**Affiliations:** 1 Danube University Krems, Center for Regenerative Medicine, Dr. Karl-Dorrek-Str. 30, 3500 Krems, Austria; 2 Karl Landsteiner University of Health Sciences, Department for Orthopedics and Traumatology, University Hospital Krems, Dr. Karl-Dorrek-Straße 30, 3500 Krems, Austria; 3 Medical University of Vienna, High-Field MR Center, Department of Biomedical Imaging and Image-Guided Therapy, Währinger-Gürtel 18-20, 1090 Vienna, Austria; 4 Department of Health and Social Sciences, St. Poelten University of Applied Sciences, Campus-Platz 1, 3100 St. Poelten, Austria; 5 ImageBiopsy Lab GmbH, Zehetnergasse 6/2/2, 1140 Vienna, Austria

**Keywords:** artificial intelligence, knee osteoarthritis, knee radiographs, clinical severity scores

## Abstract

Background: Radiographic knee osteoarthritis (OA) severity and clinical severity are often dissociated. Artificial intelligence (AI) aid was shown to increase inter-rater reliability in radiographic OA diagnosis. Thus, AI-aided radiographic diagnoses were compared against AI-unaided diagnoses with regard to their correlations with clinical severity. Methods: Seventy-one DICOMs (m/f = 27:42, mean age: 27.86 ± 6.5) (X-ray format) were used for AI analysis (KOALA software, IB Lab GmbH). Subjects were recruited from a physiotherapy trial (MLKOA). At baseline, each subject received (i) a knee X-ray and (ii) an assessment of five main scores (Tegner Scale (TAS); Knee Injury and Osteoarthritis Outcome Score (KOOS); International Physical Activity Questionnaire; Star Excursion Balance Test; Six-Minute Walk Test). Clinical assessments were repeated three times (weeks 6, 12 and 24). Three physicians analyzed the presented X-rays both with and without AI via KL grading. Analyses of the (i) inter-rater reliability (IRR) and (ii) Spearman’s Correlation Test for the overall KL score for each individual rater with clinical score were performed. Results: We found that AI-aided diagnostic ratings had a higher association with the overall KL score and the KOOS. The amount of improvement due to AI depended on the individual rater. Conclusion: AI-guided systems can improve the ratings of knee radiographs and show a stronger association with clinical severity. These results were shown to be influenced by individual readers. Thus, AI training amongst physicians might need to be increased. KL might be insufficient as a single tool for knee OA diagnosis.

## 1. Introduction

Knee and hip osteoarthritis (OA) are a leading cause of disability, with an increased incidence amongst the elderly [1]. OA is associated with pain and a decrease in mobility, which leads to a significant socioeconomic burden [1,2].

OA prevalence is expected to increase in comparison to previous decades due to an ageing and increasingly obese population [3]. Besides age and obesity, osteochondral lesions (OCLs) contribute to joint degeneration and OA [4].

Early and accurate OA diagnosis is key to preventing disease progression and optimizing treatment regimes [5]. Clinical assessment of OA is the primary diagnostic tool, whereas imaging may be overused [3].

Nevertheless, imaging modalities such as magnetic resonance imaging (MRI), computer tomography (CT) and ultrasound play a major role in OA diagnosis. Additionally, other modalities, such as vibro- and phonoarthrography, are available as alternative diagnostic tools. Magnetic resonance imaging is the most commonly used imaging modality to assess OCLs [6]. However, MRI was shown to underestimate the extent of osteochondral lesions [7,8]. Thus, MRI may lead to underdiagnosing OCLs. MRI and PET-MRI also play a distinct role in OA research, where “premorphologic” changes in cartilage can be visualized [9]. These advanced imaging techniques can also help to detect early OA and boost the understanding of structure-modifying therapies [10]. Advances in MRI technology have helped to shape a new understanding of OA as a multi-tissue disease involving not only cartilage but also bone and soft-tissue structures [11].

Vibroarthrography was shown to have a high accuracy in distinguishing healthy cartilage from damaged cartilage [12,13]. It is a non-invasive procedure and a viable tool to supplement OA diagnosis, especially if standardized protocols are being utilized [12,13].

Radiographic OA diagnosis via X-rays of the knee is still the most applied imaging technique to supplement clinical examination [10]. It is usually performed by using semi-quantitative grading scales, of which the Kellgren–Lawrence (KL) score is most commonly used [14,15]. The KL score has been defined by the World Health Organization to be the standard grading scale in OA studies. The KL displays OA severity on a five-point scale (0 = no OA sign; 4 = end-stage OA) by assessing four radiological features (osteophytes, sclerosis, joint-space-narrowing and deformity).

The KL core has been shown to have limitations: The subjective assessment of physicians in combination with vaguely defined features at each OA progression level are amongst main points of critique [16,17,18]. This subjectivity leads to poor inter-observer reliability [19,20]. The problem of less consensual assessments of radiographs is even more evident in early osteoarthritis, where prevention would still be feasible.

An attempt to deal with this flaw was undertaken by the Osteoarthritis Research Society International (OARSI) by publishing a reference atlas for OA stages in which examples for each classification stage are presented to minimize inter-observer variability due to subjective assessments [21]. However, further studies showed that the above mentioned limitations were not solved to a satisfying degree [19,20].

The consequences of poor inter-observer variability are two-fold, affecting (i) clinical assessments as well as (ii) scientific results. With regard to (i), it may lead to misdiagnosis, including a variability of diagnoses in the same patient, and unnecessary examinations, with radiation exposure and psychological stress for patients [22]. With regard to (ii), the results of trials are less comparable, with varying rates of incidences, decreased power to detect clinically relevant differences and other issues [23].

These factors are likely to contribute to the low correlation of radiological and clinical severity that has been repeatedly shown [24].

Artificial intelligence (AI) aid may be used as a strategic element to manage those limitations in radiographic OA diagnosis. AI, especially deep learning, has been shown to be efficient at recognizing patterns [25]. In imaging applications, AI can provide recommendations and aid for the radiological assessment of images. Nehrer et al., demonstrated that that AI-aided radiograph assessment of OA knees led to increased consistency between physicians, as well as to increased accuracy [26].

To our knowledge, this is the first study that investigates the correlation of clinical severity scores and radiological severity scores (KL) with and without AI aid.

The rationale of investigating the potential advantages in correlations of clinical and radiological severity scores with AI aid was to (i) provide exact diagnoses for patients that more accurately reflected their condition and thereby reduce patients’ psychological stress and (ii) lead to more comparable and relevant results for trials. Taken together, a higher correlation of clinical and radiological OA severity with AI aid could increase the standard of care in an individualized treatment setting.

The study at hand compares the correlations of unaided ratings with clinical severity against correlations of AI-aided ratings with clinical severity.

## 2. Materials and Methods

### 2.1. Study Design and Patient Selection

Table 1 gives an overview of the study design, timeline and workflow.

The MLKOA (NCT04445350) physiotherapy trial was conducted, and 69 subjects (m/f = 27:42, mean age: 27.86 ± 6.5) were included. Subject selection was performed according to the study protocol. Signed informed consent was received from each participant. Table 2 displays the exclusion and inclusion criteria for patient selection.

At baseline, a standard AP X-ray of the study knee(s) was performed, as well as baseline assessment of clinical scores and tests. Afterwards, subjects were assigned to the treatment arms of the MLKOA physiotherapy trial. In total, five different tests (Knee Injury and Osteoarthritis Outcome Score, Tegner Activity Score, Star Excursion Balance Test, Six-Minute Walk Test and International Physical Activity Questionnaire) were conducted at baseline (measurement (M)1) and at the three following time points (M2 = 6 weeks, M3 = 12 weeks, M3 = 24 weeks).

In parallel, a physician reader study was conducted:

Of all included subjects, DICOMs (X-ray format) from 46 patients, resulting in 71 X-rays of study knees, were received in a pseudonymized manner from the MLKOA Data manager for AI analysis.

Three physicians (two orthopedic surgeons (MOLU and NEMA) and one radiologist specialized in musculoskeletal radiology (RAMA)) were chosen to analyze the presented DICOM X-rays with and without AI aid. Each physician had more than 5 years of clinical experience in the field.

Physicians were asked to assess each X-ray via the KL score. To ensure consistency in the readers’ methodology, physicians were instructed before the first reading and provided with an exemplary X-ray as well as with the table below (Table 3). Briefly, physicians were asked to assign a point value to each of the four KL sub-scores (osteophytes, joint-space narrowing, sclerosis and deformity) in a semiquantitative manner. The resulting cross-sum defined the KL score.

**Figure 1:**
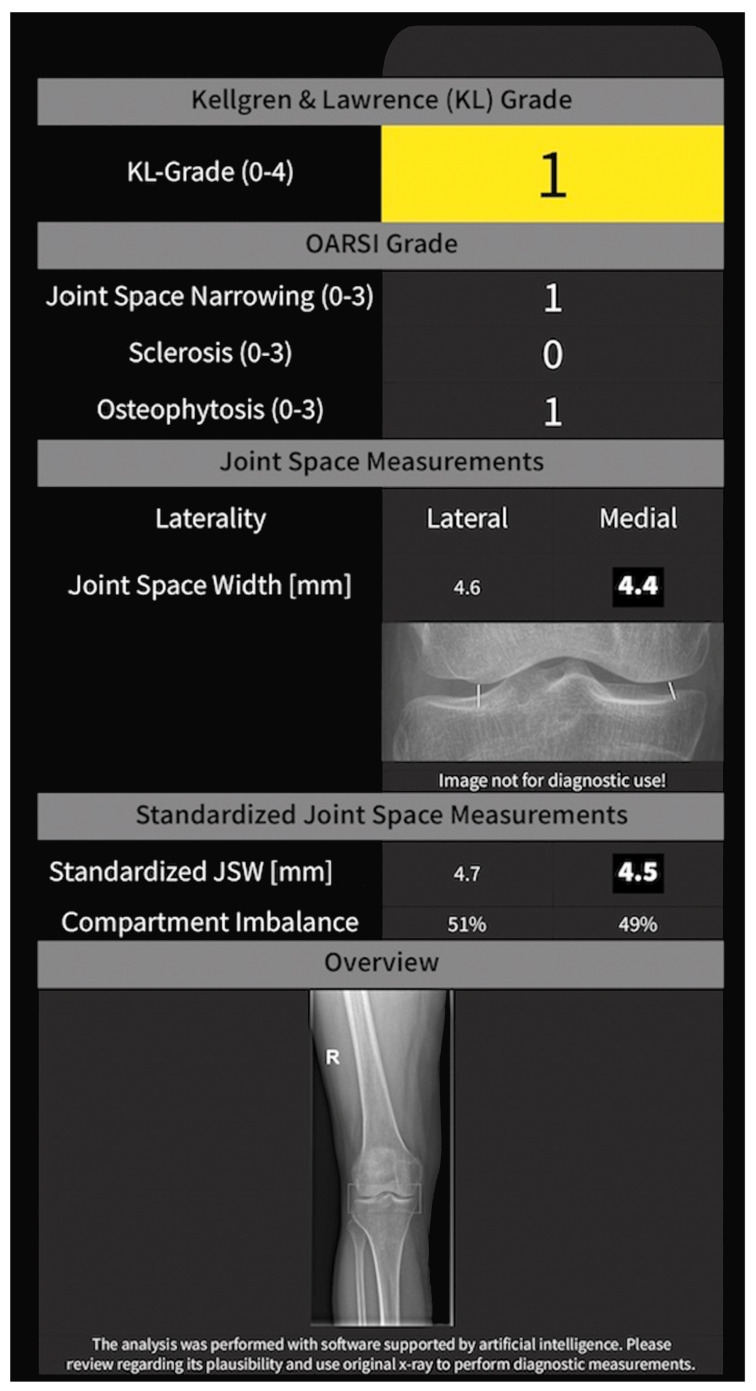
Exemplary AI-KOALA printed report.

Initially, readers were presented with X-rays without AI aid (=AI-unaided). Secondly, readers were presented with the same X-rays (in another random order) with AI aid (=AI-aided). “AI aid” was defined as a regular X-ray reading by the individual physician as described above together with a printed report (Figure 1 shows an example) provided for each X-ray. This report was created by the AI system software automatically. In this manner, the final rating decision was made by the individual physician.

### 2.2. AI System

The KOALA system is a computer-assisted detection system (KOALA, IB Lab GmbH). It was trained in a large dataset of radiographs from the “Osteoarthritis Initiative” graded for KL, JSN, sclerosis, osteophytes and OARSI grades through a consensus procedure. KOALA is based on deep learning networks to provide automated KL and OARSI grades for one printout per radiograph (Figure 1).

For the two orthopedic physicians (MOLU and NEMA), readings were performed twice-both for AI-unaided and AI-aided readings. Thus, a total of 10 independent readings (5 AI-unaided and 5 AI-aided) were performed.

Before each reading, the DICOM X-ray sequence was randomly re-ordered. There was a time period of 3 weeks in between each reading. Data were presented to readers in a pseudonymized fashion. Readers were blinded to each other’s results. Data order, presentation to readers, re-collection and re-matching with initial study IDs was performed by the IB Lab data manager.

### 2.3. Correlation Analysis with Clinical Scores

In total, 5 clinical scores—TAS, SMWT, SEBT, IPAQ and KOOS—were correlated with the overall KL score for each of the 3 raters (MOLU, NEMA and RAMA). Additionally, the subscales comprising the KL score—osteophytes, sclerosis, joint-space narrowing and deformity—were correlated with each clinical score in the same manner.

In the case of KOOS, each KOOS sub-score—activities of daily living, pain, quality of life, sports and symptoms—was independently analyzed.

### 2.4. KOOS

The Knee Injury and Osteoarthritis Outcome Score (KOOS) was developed in the 1990s. It assesses the patient’s opinion on their knee and associated limitations. It is a reliable, valid tool that has been widely evaluated and compared to other instruments [27].

It consists of 5 sub-scores: pain, symptoms, activities of daily living (ADL), function in sports and knee-related quality of life (QoL).

KOOS has been shown to have a high test–retest reliability [28].

The KOOS score with all sub-scores was calculated according to the common “KOOS scoring instructions” [29].

### 2.5. TAS

The Tegner Activity Score (TAS) was developed by Tegner et al., and is a standardized method utilizing a one-item score [30]. This score grades activity based on questions regarding sport activities on a scale of 0–10. Ten represents the best score. The score is commonly used to assess knee function.

### 2.6. SEBT

The Star Excursion Balance Test (SEBT) is a measurement tool for dynamic balance which is commonly used to measure deficits of postural control. Therefore, it is often used to measure results of rehabilitative therapies. The test has been shown to have a high reliability [31].

### 2.7. SMWT

The Six-Minute Walk Test (SMWT) is used to assess the exercise capacity of study subjects. Therefore, the test measures the distance a study subject can walk in six minutes on a pre-defined hard, flat surface. It is a commonly used tool due to its easy application and reproducibility [32].

### 2.8. IPAQ

The International Physical Activity Questionnaire (IPAQ) was developed by an international consensus group initiated by Michael Booth in Geneva, Swiss, (1998) to gather comparable data for the movement behavior of a population. The questionnaire comprises 8 instruments that assess a respondent’s movement behavior over the past week. A total of 150 min physical activity per week is considered as a threshold for sufficient activity. It has been shown that IPAQ is a reliable tool [33].

### 2.9. Statistics

Patient data are presented as mean and standard deviation. We used the irrCAC package (v1.0) to calculate Gwet’s AC2 as a measurement of inter-rater reliability. Gwet’s AC2 is superior to other methods (such as ICC) for ordinal data [34].

We calculated Spearman rank correlation with the package DescTools. The 95% confidence intervals of the correlation coefficients of each individual combination between rater, measure and time point of measurement are displayed graphically. All analyses were conducted within the R environment (v.4.1.3) (R Core Team, 2022) [35].

We used Fisher Z-transformed correlations to calculate the mean correlations. The reported values were then back-transformed with the inverse Fisher Z transformation.

## 3. Results

### 3.1. Inter-Rater Reliability

IRR was tested for the overall KL score as well for each subdomain (osteophytes, sclerosis, JSN, deformity).

IRR increased for the overall KL score with AI aid as well as for each subdomain. Confidence intervals did not overlap in any case.

**Figure 2:**
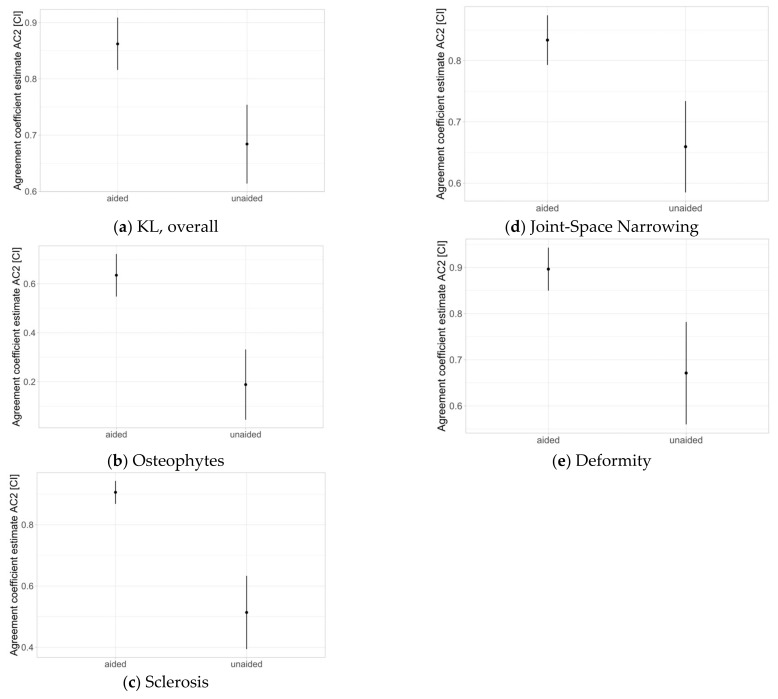
Inter-rater reliability (IRR) for the overall KL (**a**) score, as well for each subdomain (osteophytes (**b**), sclerosis (**c**), joint-space narrowing (**d**), deformity (**e**)); AC2 = agreement coefficient estimate; CI = confidence interval.

Figure 2 displays these results graphically. The numerical values of IRR are displayed in Table 4 (percentage agreement, percentage chance agreement, agreement coefficient estimate and confidence interval).

### 3.2. Mean Correlations

The mean correlation of the overall KL score with AI aid was −0.207. The mean correlation of the overall KL score with the AI-unaided approach was calculated to be −0.158. These results, as well as the subsequent correlations for each sub-scale, are presented in Table 5.

The results below are presented in sections, starting with the overall KL score and followed by its sub-scores. Each section follows the same sequence, starting with the results from each clinical score, the baseline M1 measurement, the rater(s) and finally whether or not the results were consistent over time (M2-M4).

### 3.3. Overall KL

SMWT, SEBT: No relevant difference in AI-unaided versus AI-aided correlations was detected.

TAS: At the M1, NEMA1 and NEMA2 showed a superior inverse correlation in favor of AI aid. This trend was enhanced and consistent over time (M2-M4).

IPAQ: At every measurement time point, and consistent with every reader, IPAQ showed a positive correlation without relevant differences between AI-aided/-unaided correlations.

KOOS: For all five sub-scores (activities of daily living, pain, quality of life, sports and symptoms) at the M1 baseline measurement, a superior inverse correlation in favor of AI aid was shown for MOLU1+2 and NEMA1+2. In the case of RAMA, the same finding was shown only for KOOS sub-scores of QoL at M1.

**Figure 3:**
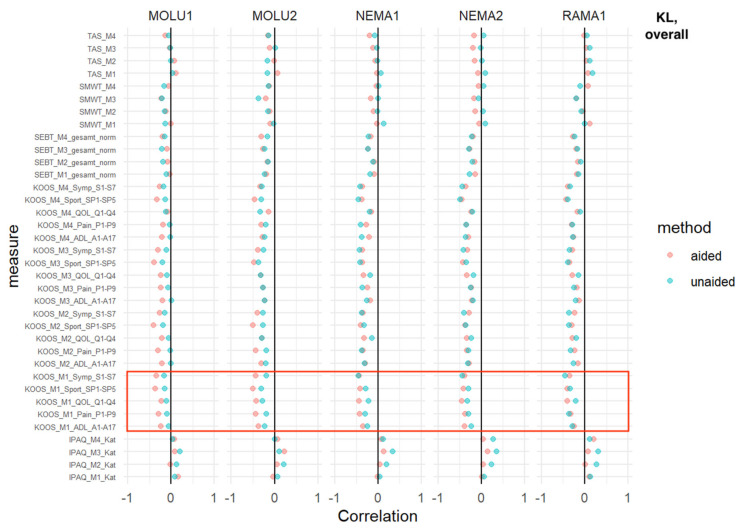
*Overall KL*—correlation analysis for AI-aided (red dots) versus AI-unaided (green dots) readings with clinical scores. Red square marks all baseline KOOS scores (=“KOOS_M1_x_x”). **The abbreviations used are the same in**Figure 3, Figure 4, Figure 5, Figure 6 and Figure 7**.** MOLU1+2; NEMA1+2; RAMA = physician readers; “Mx” = measurement. M1 = baseline, M2 = 6-week follow-up, M3 = 12-week follow-up, M4 = 24-week follow-up. IPAQ_Mx_Kat = International Physical Activity Questionnaire; KOOS_Mx_x-subscore_numbers of questions in questionnaire, KOOS = Knee Injury and Osteoarthritis Outcome Score. Five sub-scores: ADL = activities of daily living; pain = pain; QoL = quality of life; sport = sport; Symp = symptoms. SEBT_Mx_ges_norm = Star Excursion Balance Test; SMWT_Mx = Six-Minute Walk Test; TAS_Mx = Tegner Activity Score.

This trend was consistent for MOLU1 in most KOOS sub-scores for M2-M4 (Figure 3).

### 3.4. Osteophytes

TAS, SMWT, SEBT: No relevant difference in AI-unaided versus AI-aided correlations was detected.

IPAQ: At every measurement time point, and consistent with every reader, IPAQ showed a positive correlation without relevant differences between AI-aided/-unaided correlations.

KOOS: For all five sub-scores (activities of daily living, pain, quality of life, sports and symptoms) at the M1 baseline measurement, a superior inverse correlation in favor of AI aid was shown for MOLU1+2.

**Figure 4:**
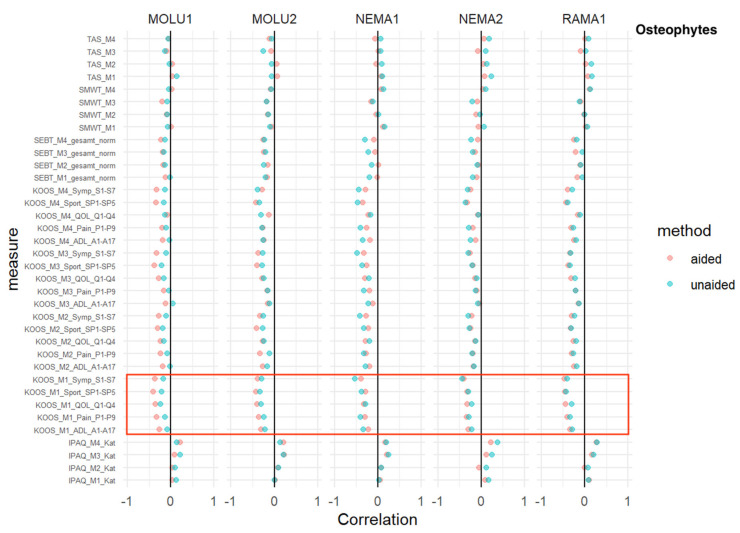
*Osteophytes*—correlation analysis for AI-aided (red dots) versus AI-unaided (green dots) readings with clinical scores. Red square marks all baseline KOOS scores (=“KOOS_M1_x_x”).

This trend was consistent for MOLU1 in every KOOS sub-score for M2-M4, except for the domain “pain”, where the trend of inverse correlation was only seen for M1+M2 (Figure 4).

### 3.5. Sclerosis

TAS, SMWT, SEBT: No relevant difference in AI-aided versus AI-aided correlations was detected.

IPAQ: At every measurement time point, and consistent with every reader, IPAQ showed a positive correlation without relevant differences between AI-aided/-unaided correlations.

KOOS: For three sub-scores (pain, quality of life and sports) at the M1 baseline measurement, a superior inverse correlation in favor of AI aid was shown for MOLU1+2 and NEMA1.

**Figure 5:**
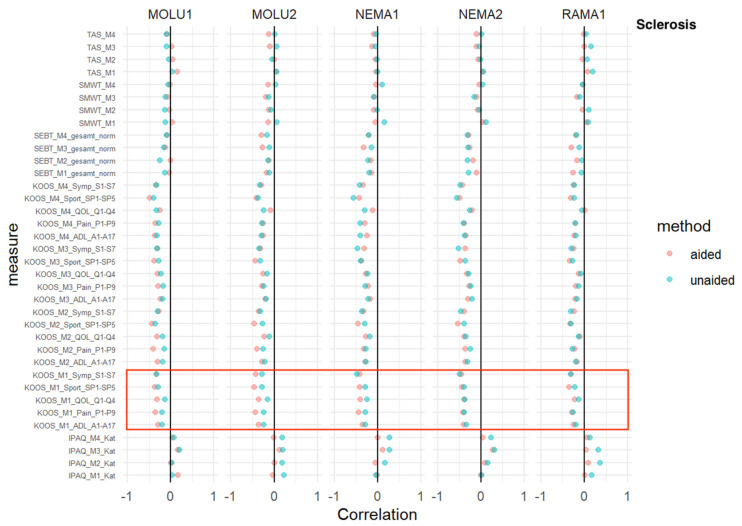
*Sclerosis*—correlation analysis for AI-aided (red dots) versus AI-unaided (green dots) readings with clinical scores. Red square marks all baseline KOOS scores (=“KOOS_M1_x_x”).

This trend was consistent for MOLU1+2 for two sub-scores (pain and quality of life) at M2 (Figure 5).

### 3.6. Joint-Space Narrowing

TAS, SMWT, SEBT: No relevant difference in AI-aided versus AI-aided correlations was detected.

IPAQ: At every measurement time point, and consistent with every reader, IPAQ showed a positive correlation without relevant differences between AI-aided/-unaided correlations.

For two sub-scores (pain and ADL) at the M1 baseline measurement, a superior inverse correlation in favor of AI aid was shown for MOLU1+2.

**Figure 6:**
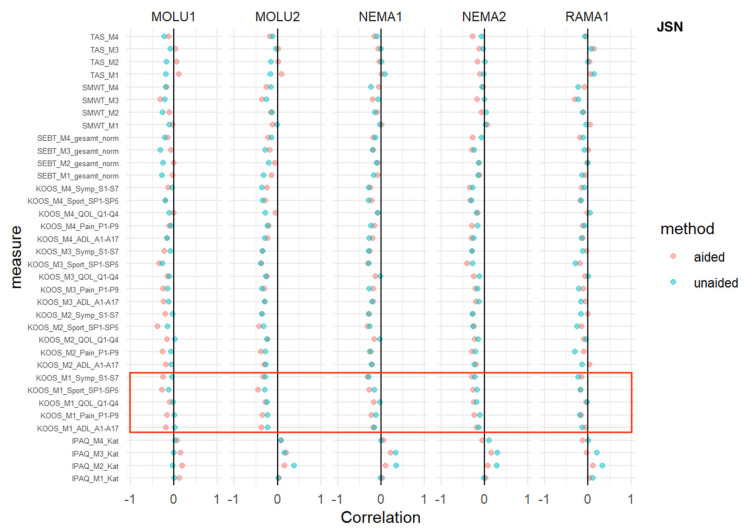
*Joint-space narrowing (JSN)*—correlation analysis for AI-aided (red dots) versus AI-unaided (green dots) readings with clinical scores. Red square marks all baseline KOOS scores (=“KOOS_M1_x_x”).

This trend was consistent for MOLU1 for one sub-score (pain) at M2 (Figure 6).

### 3.7. Deformity

TAS, SMWT, SEBT, KOOS: No relevant difference in AI-aided versus AI-aided correlations was detected at baseline M1.

**Figure 7:**
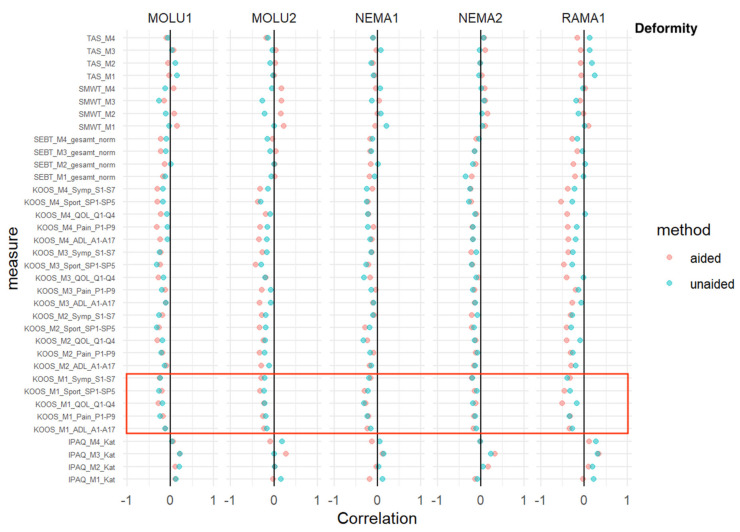
*Deformity*—correlation analysis for AI-aided (red dots) versus AI-unaided (green dots) readings with clinical scores. Red square marks all baseline KOOS scores (=“KOOS_M1_x_x”).

IPAQ: At every measurement time point, and consistent with every reader, IPAQ showed a predominantly positive correlation without relevant differences between AI-aided/-unaided correlations, as shown in Figure 7.

### 3.8. Key Findings

Most absolute correlation coefficients between ratings and clinical severity were below 0.5 (Figure 3, Figure 4, Figure 5, Figure 6 and Figure 7), regardless of AI guidance. Correlation coefficients varied between different outcomes, raters and measures. Aside from specific combinations of outcomes, measures and raters, the correlation coefficients were quite similar.

For the overall KL, a weak trend of superior inverse correlation in favor of AI aid was shown for (i) TAS, as well as (ii) KOOS and all KOOS sub-scores in two raters, and in one sub-score (QoL) at baseline measurement M1 for all three raters.

A similar trend of superior inverse correlation in favor of AI aid was shown in the case of osteophytes > (less) for sclerosis > and the least for JSN. When analyzing KL subscales, no relevant difference between AI-aided and AI-unaided readings was shown for “deformity”.

The quantity of reads with superior inverse correlation in favor of AI aid differed between raters, with MOLU1+2 having the most, NEMA1+2 less and RAMA the least.

IPAQ showed a predominantly positive correlation without relevant differences between AI-aided/-unaided correlations consistently for all raters and all scores.

Other scores did not show relevant differences in AI-aided versus AI-aided correlations.

Additionally, Tables S1–S5 (KL grade, JSN, osteophytes, sclerosis and deformity) are provided as Supplementary Materials, displaying the numerical data behind the presented graphs as well as the confidence intervals.

## 4. Discussion

The aim of this study was to compare the association of clinical severity scores and radiological severity scores (KL) with AI aid versus a standard AI-unaided assessment.

The three key findings of this study may point to the following context within knee OA diagnosis and treatments:

1. AI-aided diagnostic ratings have a higher association with the overall KL score and the KOOS score.
2. The amount of the improvement depends on the individual rater.
3. The KL score might be insufficient as a single tool for knee OA diagnosis.

Our findings indicate a poor association of the KL score with clinical severity scores. This association did slightly improve with AI aid. However, the KL score still appears to be a weak tool with little association between clinical severity and radiographic ratings.

A possible explanation for the higher association of clinical and radiological OA severity in the case of AI aid is the increased consistency between physicians and the increased accuracy that was shown for AI-aided OA radiographic diagnosis [26].

The KL score is widely used. Epidemiological landmark studies, such as Felson et al., and the Framingham Osteoarthritis Study, based their findings on the KL score [36,37]. The KL score is also a widely used tool in the clinical assessment of knee OA [14].

The common and known dissociation of clinical symptoms and radiological OA severity [38] has been a major issue in (i) OA studies for reasons of intra- and inter-study comparability reducing statistical power [39], as well as for (ii) individualized, precise OA treatments. This leads to misdiagnosis, psychological stress for patients and omitted treatments [22].

Thus, AI aid in knee OA diagnosis may help to tackle the abovementioned issues and increase the standard of knee OA care.

Other groups have also applied deep learning techniques to assess OA in knee radiographs using the KL score [40]. Abdullah et al., demonstrated that the fine tuning of networks increased the performance of AI-based OA diagnosis via the KL score [40]. However, automated OA diagnosis remains troublesome, and extensions to MRI based approaches are warranted [40]. Deep collaborative network approaches are already being investigated in MRI OA diagnosis and are likely to become more prominent, especially in OA research [41,42]. A trend towards automated OA diagnosis via MRI seems to be likely when considering the weaknesses of currently used radiographic grading tools such as the KL score, which are also shown in the presented work.

Research is warranted to reduce the weaknesses of deep learning models to routinely use promising AI-aided OA diagnostic tools [43]. However, as this study demonstrates, it is not only the machine learning but also the underlying scoring system that may need improvement. Nevertheless, recent breakthroughs in deep learning and AI applications for OA diagnosis exhibit the potential for soon-to-be routine clinical use that helps to diagnose and predict the course of the disease [44].

To our knowledge, this is one of the first papers investigating the association of clinical severity and radiological severity with AI aid.

The variety of superior inverse correlations with clinical scores in favor of AI with regard to the specific AI subscales is an intriguing finding, with “deformity” having no relevant correlation and “osteophytes” having the most explicit association. This finding supports the consensus in the orthopedic scientific community that criticizes the limitations of the old but often-used KL score. Moreover, these findings support efforts to create a novel grading system. However, it may be that another, easier-to-handle synthesized version of the KL score, which combines OARSI scoring and KL scoring without the subscale of “deformity”, is desirable [45].

Our study population and data were drawn from a physiotherapy trial, which is a limiting factor. Another limiting factor is that the MSK radiologist RAMA only completed one read with and without AI aid.

In order to confirm these findings, subsequent studies with prospective designs are warranted.

It appears that physician raters are unequally influenced by AI. Interestingly, the MSK radiologist RAMA showed the least correlation with AI. As skepticism towards AI aid for radiologists has been described previously [46], this may contribute to the presented findings. Thus, interestingly, physicians’ psychological factors seem to play a role in AI acceptance and thus usability. AI–physician education, where the role of physicians is both appreciated and adapted together with sound data-based information about the distinct advantages of AI aid in diagnostics, is a strategy that can potentially be implemented in physicians’ continued education [47].

The abovementioned factors may help to reduce the OA burden by more accurate detection, which is a major goal of the orthopedic community as a whole [48].

## 5. Conclusions

A broad consensus in the scientific orthopedic community states that a reduction in the OA disease burden is pivotal. A major pillar to achieving this goal is accurate, early diagnosis.

The presented data show that AI aid improves the association of radiographic ratings with clinical severity in knee OA. However, the KL score still appears to be a weak assessment tool.

A novel grading scale with AI aid is potentially necessary to meet the abovementioned aims of accurate OA diagnosis.

Radiologists and physicians in general need to be educated and informed about how to properly implement AI aid in a co-operative manner.

## Figures and Tables

**Table 1:** Study design, timeline and workflow (AI = artificial intelligence; ff = following; M = measurement; w = week. “X” marks when outlined task was conducted; → = arrow indicates “new random order of DICOMs in between readings”).

		Part A: Active Trial				Part B: Physician Reader Study					Correlation Analysis
Inclusion		M1	M2	M3	M4	ff M4	+3w		+3w	+3w	
	X-ray	X									
	Clinical Score	X	X	X	X						ff
	AI-Unaided					X→X					
	AI Analysis							X			
	AI-Aided								X→X		
	Start	01/2019				11/2021					06/2022
	End				10/2021					06/2022	09/2022
Timeline 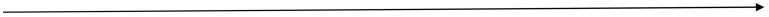											

AI = artificial intelligence; ff = following; M = measurement; w = week. “X” marks when outlined task was conducted; → = arrow indicates “new random order of DICOMs in between readings”.

**Table 2:** Inclusion and exclusion criteria for patient selection.

Inclusion Criteria	Exclusion Criteria
Kellgren–Lawrence score 1–3	Activated knee OA
BMI < 33	Lower extremity surgery in the past 6 months
Free range of motion in the knee joint	Intake or injection of corticosteroids in the past 3 months
	Long-term NSAR medication
	Neurological disease Drug or alcohol abuse Post-traumatic OA

BMI = body mass index; NSAR = non-steroidal anti-inflammatory drugs; OA = osteoarthritis.

**Table 3:** KL semi-quantitative assessment: point scores and descriptions (JSN = joint-space narrowing; KL = Kellgren–Lawrence; OA = osteoarthritis).

Parameter	Assessment	Point Value	KL Score	KL Description
osteophytes	none definite large	0 1 2		
JSN	no narrowing/doubtful definite JSN extreme JSN no more space/bone on bone	0 1 2 3		
sclerosis	none mild mild + cysts strong + cysts	0 1 2 3		
deformity	none mild strong	0 1 2		
sum total		0	0	no OA sign
		1–2	1	slight sclerosis or osteophytes
		3–4	2	slight JSN + osteophytes
		5–9	3	definite osteophytes + JSN
		10	4	end-stage OA

**Table 4:** Numerical values of inter-rater reliability analysis (AC2 = agreement coefficient estimate; CI = confidence interval; PA = percentage agreement; PCA = percentage chance agreement).

	KL		Osteophytes		Sclerosis		JSN		Deformity	
	Unaided	Aided	Unaided	Aided	Unaided	Aided	Unaided	Aided	Unaided	Aided
**PA**	89.67%	95.49%	72.95%	87.68%	83.25%	96.03%	87.87%	93.67%	85.51%	93.24%
**PCA**	67.3%	67.28%	66.66%	66.24%	65.56%	57.82%	64.37%	61.94%	55.91%	34.61%
**AC2**	0.68	0.86	0.19	0.64	0.51	0.91	0.66	0.83	0.67	0.9
**CI**	0.614:0.754	0.816:0.909	0.45:0.332	0.548:0.72	0.394:0.633	0.868:0.943	0.585:0.734	0.793:0.874	0.56:0.782	0.85:0.943

**Table 5:** Mean correlations of the overall KL score and its sub-scores (JSN = joint-space narrowing; KL = Kellgren–Lawrence).

Mean Correlation					
	KL, Overall	Osteophytes	Sclerosis	JSN	Deformity
**aided**	−0.207	−0.163	−0.207	−0.141	−0.142
**unaided**	−0.158	−0.136	−0.163	−0.125	−0.103

## Data Availability

Data available on request due to restrictions, e.g., privacy or ethical. The data presented in this study are available on request from the corresponding author.

## Supplementary Materials

The following supporting information can be downloaded at: https://www.mdpi.com/article/10.3390/jcm12030744/s1, Five tables (KL grade, JSN, osteophytes, sclerosis and deformity) are provided as Supplementary Materials, displaying the numerical data behind the presented graphs as well as the confidence intervals. (Table S1: Deformaty; Table S2: JSN (Joint Space Narrowing); Table S3: KL-Grade; Table S4: Osteophytes; Table S5: Sclerosis).
